# Constructing Brain Connectivity Model Using Causal Network Reconstruction Approach

**DOI:** 10.3389/fninf.2021.619557

**Published:** 2021-02-18

**Authors:** Supat Saetia, Natsue Yoshimura, Yasuharu Koike

**Affiliations:** ^1^Department of Information Processing, Interdisciplinary Graduate School of Science and Engineering, Tokyo Institute of Technology, Yokohama, Japan; ^2^Precursory Research for Embryonic Science and Technology (PRESTO), Japan Science and Technology Agency (JST), Kawaguchi, Japan

**Keywords:** fMRI, connectivity, causality, brain, motor

## Abstract

Studying brain function is a challenging task. In the past, we could only study brain anatomical structures post-mortem, or infer brain functions from clinical data of patients with a brain injury. Nowadays technology, such as functional magnetic resonance imaging (fMRI), enable non-invasive brain activity observation. Several approaches have been proposed to interpret brain activity data. The brain connectivity model is a graphical tool that represents the interaction between brain regions, during certain states. It depicts how a brain region cause changes to other parts of the brain, which can be implied as information flow. This model can be used to help interpret how the brain works. There are several mathematical frameworks that can be used to infer the connectivity model from brain activity signals. Granger causality is one such approach and is one of the first that has been applied to brain activity data. However, due to the concept of the framework, such as the use of pairwise correlation, combined with the limitation of brain activity data such as low temporal resolution in case of fMRI signal, makes the interpretation of the connectivity difficult. We therefore propose the application of the Tigramite causal discovery framework on fMRI data. The Tigramite framework uses measures such as causal effect to analyze causal relations in the system. This enables the framework to identify both direct and indirect pathways or connectivities. In this paper, we applied the framework to the Human Connectome Project motor task-fMRI dataset. We then present the results and discuss how the framework improves interpretability of the connectivity model. We hope that this framework will help us understand more complex brain functions such as memory, consciousness, or the resting-state of the brain, in the future.

## 1. Introduction

According to the theory of evolution, animals, including humans, as we know them today started from simple organisms, then evolved and diverged over time into several species of animals. Some animals have what looks like simplistic behavior, and some are more complicated than others. Complex behavior in an animal is governed by the development of its central nervous system. Earthworms have a relatively simple nervous system, and their behaviors reflect that—they are observed to behave according to their set of innate behaviors (McManus et al., 1982). Animals with a more developed central nervous system, some birds for instance, exhibit more complex future-oriented behavior, such as food caching in anticipation of upcoming environmental changes (Roth, 2015). Animals like dogs show higher cognitive function with their social behavior among other dogs and even with humans. Even more advanced animals, namely elephants (Plotnik et al., 2006) and dolphins (Reiss and Marino, 2001), exhibit even higher cognitive function, such as self-recognition of their own reflection when presented with a mirror, which is a rare cognitive capability in the animal kingdom.

At the apex of that central nervous system development is humankind. We are capable of performing complex cognitive tasks that no other animals have been observed to be capable of. Our perceptions enable us to recognize and interpret sensory stimuli, process the meaning of what we see, hear, feel, and taste, and then storing these stimuli in our memory. Our linguistic abilities enable the transfer of knowledge or ideas. The ability to spontaneously perform memory-related cognitive functions is theorized to be a basis of higher cognitive function, such as problem-solving, decision-making, and even the theory of mind, which is a theory about how humans are able to recognize their own selves and project that self within their mind, resulting in the individuality of each human (Schacter et al., 2015).

The brain is at the center of the central nervous system. Understanding its mechanism is important to understanding how all aforementioned cognitive functions work. Studying brain mechanisms presents several difficulties, mainly the fact that brain activity cannot be physically observed from the outside. In the past, it was difficult to observe the brain activity of a living subject without the risk of an invasive medical procedure. Nonetheless, post-mortem studies of brain cellular structures have provided us with an idea of how the brain is anatomically organized and, combined with medical and psychological observation of patients with specific cognitive prognosis or brain injuries, several studies have aimed to interpret how brain mechanisms work based on those observations.

Current technological advances such as electroencephalography (EEG) and functional magnetic resonance imaging (fMRI) techniques enable us to observe live brain activities non-invasively using external sensors. Each technique has its own advantages and disadvantages. The EEG technique yields a high temporal resolution signal because it monitors brain electrical activity, however, its signal has low spatial resolution since the recorded electrical brain activity is observed through the layers of the skull and scalp. Functional magnetic resonance imaging (fMRI) is an extension of the MRI technique, which is an imaging technique developed to form an image of an internal anatomical structure without the need of an invasive procedure. MRI scanners use strong magnetic fields to polarize and then detect hydrogen atoms, abundantly present in the human body in the form of water and fat, to construct anatomical structure images. The fMRI technique extends conventional MRI by observing blood flow or blood-oxygen-level-dependent (BOLD) through the brain over time. We can infer brain activity from the BOLD signal on the assumption that the active brain areas consume energy, thus require relatively higher amounts of blood in comparison to the inactive areas. The fMRI technique yields high spatial resolution in terms of localization of the active brain area; however, it has low temporal resolution due to the fact that it observes the physical displacement of blood, which is relatively slow compared to the actual underlying electrical neuronal activity.

With the current technology, we can observe live brain activity. The next question is how we would interpret this information. Conventional fMRI studies can show us the localization of brain activity in relation to the cognitive function of interest. Knowing which area of the brain is active during which cognitive task does not provide us with a full understanding of the mechanisms of the brain. Understanding the interaction between those active brain regions will provide us with a deeper understanding of dynamic brain function. The concept of the brain connectivity model was developed to represent those interactions between brain regions. There are three types of brain connectivity models, anatomical, functional, and effective connectivity models. The anatomical connectivity model represents the actual physical connection based on the brain's cellular structure and organization (Wang et al., 2013), knowledge mostly acquired through post-mortem studies of the brain. Functional models represent an un-directed statistical relationship between brain regions, while the effective model represents a directed causal relationship between brain regions. These are usually constructed by analyzing brain activity data. We can interpret the information provided by these models to infer how brain mechanisms works.

There are several mathematical frameworks and algorithms available for functional or effective connectivity model inference from recorded brain signal time-series. One of the most well-known frameworks is Granger causality (GC), a linear auto-regressive causality modeling framework (Granger, 1969). The underlying causality definition of this framework is that *X*→*Y* if, and only if, change in *X* has an effect on *Y* (Pearl, 2009). However, this framework only measures statistical dependencies (correlation) between activities of brain regions. Moreover, in case of fMRI, the fact that BOLD time-series is only by-products of the actual neuronal signal and has low temporal resolution, further confounds the connectivity model, where the basis assumption relies on correct temporal order of the signal.

In recent years, several tools designed specifically for brain connectivity study using fMRI data, such as Dynamic Causal Modeling (DCM) (Friston et al., 2003) or CONN toolbox (Whitfield-Gabrieli and Nieto-Castanon, 2012) have been developed. Each attempts to address the aforementioned issue from different aspects. The DCM constructs the connectivity model by predicting neuronal activity using a forward model, then it uses a hemodynamic model to generate a hypothetical BOLD time-series. Finally, it tests the hypothesis against the real BOLD time-series to choose the best model. The CONN toolbox performs temporal pre-processing on BOLD time-series in addition to conventional spatial pre-processing to remove noises that usually cause spurious connection in the model as much as possible. Each approach has its own advantages and disadvantages. For example, the DCM needs a concrete assumption of the driver that causes changes in the system, and it is usually suitable with a task-based fMRI paradigm. The CONN toolbox, which is usually applied to resting-state fMRI, is only designed for functional connectivity that only shows correlation between brain regions, in contrast to the effective connectivity model which encodes more information in form of a directed link. The additional information improves the model interpretability.

In the context of fMRI analysis, DCM is a technique developed specifically for analysing connectivity from fMRI BOLD time-series and it is arguably one of the most widely adopted methods. This technique is a model-based approach where it constructs a connectivity model by simulating a hypothetical model supplemented by a hemodynamic forward model. It then estimates the model parameters from observed data (Friston et al., 2003). DCM requires *a priori* knowledge about the structure of the network being estimated to test different specific hypotheses using Bayesian model comparison. For this reason, the classical DCM is only suitable for a task-based experiment paradigm where input functions are known. To extend the capability of DCM to cover resting-state analysis where input function is not well-defined, a DCM for resting-state fMRI (spectral DCM) was developed (Friston et al., 2014). The new developed technique fits a model to the cross spectrum of the data. Cross spectra are second-order statistics of the original time-series under stationarity assumption. However, the resting-state analysis usually includes a larger number of regions of interest, which can be a challenge for DCM in terms of computational cost. Razi et al. proposes a framework complimentary to spectral DCM, using functional models as priors to reduce computational complexity of a large-scale network (Razi et al., 2017).

Tigramite (time-series graph-based measures of information transfer) is a time-lagged causal discovery framework based on conditional independence testing using the assumptions of time-order, *Causal Sufficiency*, the *Causal Markov Condition*, and *Faithfulness*, among others (Runge, 2018). The inclusion of time-lag enables this framework to show changes in the causal model over time, which is useful for pathway inference. The connectivity models can be visualized in form of *graphical model* (Lauritzen, 1996) which is a summary model showing all existing connectivities, or *time-series graph* (Eichler, 2012), a graph that shows a causal relation along a lagged-timeline. This visualization is useful for model interpretation.

The development of this framework started with an attempt to escape the curse of dimensionality in estimating multivariate transfer entropy from observational climate data (sea level pressure) (Runge et al., 2012). Transfer entropy (TE) is a model-free approach to detect directed transfer of information (causality) between a stochastic process (Schreiber, 2000). The main problems with the interpretation of causal influences in a system where the underlying mechanisms are poorly understood, are the possibility of spurious causalities from indirect influences or common drivers (Runge et al., 2012). When interpreting the relationship between two process, it can be said to be a causal relationship if a statistical methods can (1) measure associations, (2) measure time delays, and (3) exclude other influences (Pearl, 2009). An existing model-based approach such as Granger causality fulfills requirements (1) and (2). The unfulfilled requirement (3) makes it controversial to infer causal relationship in this approach. There are no such model-based requirements in the information theoretic framework (Cover and Thomas, 2006). The information-theoretic function utilized in this framework is conditional mutual information (CMI) (Hlaváčková-Schindler et al., 2007) in the form of transfer entropy (TE) (Schreiber, 2000).

The main advantage of choosing TE over conventional methods such as DCM is that it is model-free and is also capable of detecting both linear and non-linear dependencies. DCM relies on correct prior knowledge of the network under investigation to define the optimal model space, because the model space should reflect the possible causal connection between the brain region in the network (Kahan and Foltynie, 2013). Therefore, it may not be optimal for exploratory analyses. Vicente et al. formulated four requirements for a new effective connectivity measure for it to be considered a useful addition to the established methods, such as GC and DCM, and showed that TE fulfills those requirements. (Vicente et al., 2010). The four requirements are as follows:

1. It should not require the *a priori*.
2. It should be able to detect non-linear interactions.
3. It should be able to detect effective connectivity even if there is a wide distribution of interaction delays between two nodes.
4. It should be robust against linear cross-talk between signals.

The first requirement ensures that the new measure is useful for exploratory investigations. DCM was designed specifically for fMRI data by including a generative model based on hemodynamic function (Buxton et al., 1998), which can be both a strength and a weakness because, while the model fits the data well, it depends on the accuracy of the current knowledge regarding hemodynamic response (Bielczyk et al., 2019). There are new studies suggesting that hemodynamic responses vary across populations based on the physical conditions of the individuals (Handwerker et al., 2012). Nonetheless, the model-free measure could also be used in addition to DCM in large-scale analyses to create prior constraints, to reduce the model space of large networks. The second and third requirements are dictated by observed characteristics of brain function. The brain exhibits strong non-linear interactions across all levels of brain function. The signal, traveling from one brain region to another, also involves several pathways where delays could be varied according to the anatomical structure (Swadlow and Waxman, 1975). The fourth requirement ensures the quality of the analysis using signals from electroencephalography (EEG) or magnetoencephalography (MEG), however, this property could still benefit from further analysis using the fMRI signal.

There are several studies that utilize TE to investigate brain connectivity. The studies by Wibral et al. investigate brain connectivity using *multivariate transfer entropy* (Novelli et al., 2019) in both task and resting-state paradigms (Wibral et al., 2017a,b). On the other hand, the Tigramite framework proposes the use of graphical causality in combination with information theoretic measure i.e., TE. The Tigramite utilizes the PCMCI algorithm (Runge, 2018), which was proposed to address the shortcomings of the Peter and Clark (PC) algorithm (Spirtes and Glymour, 2016). The PC algorithm is a graph-based causal discovery algorithm where it starts with a complete undirected graphical model (Lauritzen, 1996), then the links are adjusted based on a conditional independence test (Spirtes and Glymour, 2016). The PCMCI algorithm has two main steps. In the first step, a version of the PC algorithm is used to estimate parent sets of each variable. Then, in the second step, it performs the *momentary conditional independence* (MCI) test for each pair of variables and conditions on the aforementioned parent sets. This reduces the number of independence tests it needs to perform. The important advantage of PCMCI over PC is that the MCI test accounts for autocorrelation which keeps the false-positive rates at the expected level (Runge et al., 2017).

This study is the first to attempt to apply the Tigramite framework to fMRI time-series. We applied the Tigramite framework to motor task-fMRI data collected and distributed by the Human Connectome Project (HCP) (Van Essen et al., 2012). Later in this paper, we discuss how we applied the framework to fMRI BOLD time-series, and how we interpret the resulting model in context of brain connectivity.

## 2. Materials and Methods

### 2.1. HCP Dataset and Protocols

**Figure 1:**
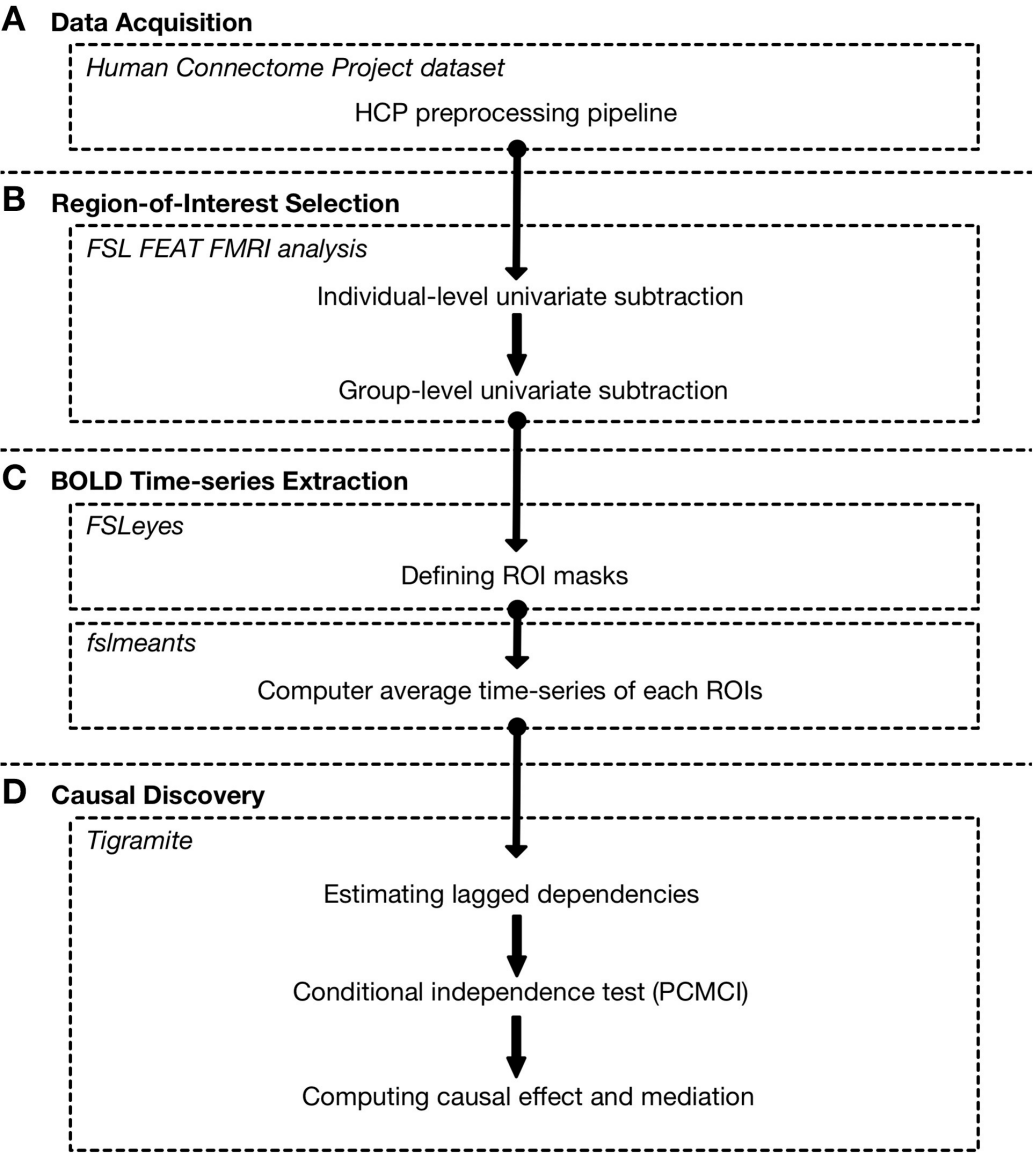
Summary of processing pipeline from fMRI data to connectivity using Tigramite. **(A)** Here, we use a motor task-fMRI dataset provided by the Human Connectome Project (HCP). The data were pre-processed by the HCP using the HCP pre-processing pipeline (Glasser et al., 2013), which includes image reconstruction, distortion correction, motion correction, and MNI nonlinear volume registration. **(B)** We performed localization of the activated brain region during the task using *FSL FEAT fMRI analysis*. Group-level analyses were done using 50 randomly selected individuals. **(C)** We then defined masks for BOLD time-series extraction based on the aforementioned localization results using *FSLeyes*. Additional regions that are known to play roles in motor-related function were included. The average BOLD time-series of each region of interest was then extracted using the *fslmeants* tool. **(D)** Finally, Tigramite was used to construct a connectivity from the extracted time-series. The first step is to estimate the lagged dependencies to determine the maximum lag (τ_*max*_). We then performed the conditional independence test to estimate the causal link between each ROI. After that, the estimated time-series graph was used to evaluate the causal effect and causal mediation.

The Human Connectome Project (HCP) is a project conducted by the Washington University-University of Minnesota Human Connectome Project Consortium (WU-Minn HCP) (Van Essen et al., 2012). This dataset provides access to exceptional spatiotemporal resolution fMRI data of a large well-characterized group of healthy individuals. We utilized HCP motor task-fMRI for this study (Figure 1A). This motor task was adapted from a design developed by Bucholz et al. (1994). Subjects were asked to perform the following actions: tapping left or right fingers, squeezing left or right toes, or moving tongue according to a visual cue presented. The session was organized in blocks of movement type, each preceded by a 3 s cue, and lasted for 12 s with 10 movements. Overall, the session contains 13 blocks in total, with four foot movements (two right and two left), four hand movements (two right and two left), and two tongue movements. The remaining three blocks are 15 s fixation blocks.

### 2.2. Univariate Group Subtraction Analysis

A univariate group subtraction analysis was performed to identify activated brain regions while the subjects were performing the tasks. The fMRI images were processed and analyzed using FSL 6.0.2 (FMRIBs Software Library, www.fmrib.ox.ac.uk/fsl) software suite (Figure 1B). The pre-processing consisted of image reconstruction, distortion correction, motion correction, and slice timing correction. The HCPs structural MRI and fMRI were pre-processed using FSL 5.0.6 according to the HCP pre-processing pipeline (Glasser et al., 2013). Individual- and group-level univariate group subtraction analyses were done using FEAT (FMRI Expert Analysis Tool, v.6.00). Fifty subjects were randomly selected from the HCP dataset for this analysis.

### 2.3. Region-of-Interest Selection and Time-Series Extraction

To construct a connectivity model, a region-of-interest (ROI) set must be selected. A ROI set was usually selected based on a univariate group subtraction analysis. Additionally, ROI can also be selected from related literature. In this study, the main effect of motor task is identified in the left and right anterior primary motor cortex Brodmann area 4, left and right posterior primary motor cortex Brodmann area 4, left and right premotor cortex Brodmann area 6, left and right visual cortex V1 Brodmann area 17, and left and right visual cortex V2 Brodmann area 18. Furthermore, the frontal lobe, cerebellum, left and right thalamus, are included based on prior studies (Manto et al., 2011) where they a play role in motor related task. The masks of the aforementioned region were created using FSLeyes software (Figure 1C). Each mask has been checked to ensure that there is no overlapping between different regions.

BOLD time-series were extracted using FSL's average time-series calculation tool (fslmeants). The time-series of each area was extracted according to the Juelich histological atlas, the Harvard-Oxford cortical structural atlas, and the MNI structural atlas (Table 1). Slice timing correction was performed using FSL.

**Table 1:** All region of interest included in connectivity model.

**No**.	**Area name**	**Abbreviation**	**Atlas**
1	Left thalamus	PmcBA4pL	MNI structural
2	Right thalamus	PmcBA4pR	MNI structural
3	Left premotor cortex Brodmann area 6	PcBA6L	Juelich histological
4	Right premotor cortex Brodmann area 6	PcBA6R	Juelich histological
5	Left anterior primary motor cortex Brodmann area 4	PmcBA4aL	Juelich histological
6	Right anterior primary motor cortex Brodmann area 4	PmcBA4aR	Juelich histological
7	Left posterior primary motor cortex Brodmann area 4	PmcBA4pL	Juelich histological
8	Right posterior primary motor cortex Brodmann area 4	PmcBA4pR	Juelich histological
9	Left visual cortex V1 Brodmann area 17	V1BA17L	Juelich histological
10	Right visual cortex V1 Brodmann area 17	V1BA17R	Juelich histological
11	Left visual cortex V2 Brodmann area 18	V1BA18L	Juelich histological
12	Right visual cortex V2 Brodmann area 18	V1BA18R	Juelich histological
13	Cerebellum	Cereb	Harvard-Oxford cortical structural
14	Frontal lobe	FL	Harvard-Oxford cortical structural

### 2.4. Tigramite Causal Discovery

For connectivity model construction, we propose the use of the Tigramite causal discovery framework (Figure 1D). To obtain causal information from measured variables, some assumptions are needed. This framework focuses on three main assumptions under which the time-series graph represents a causal relation (Runge, 2018).

The first assumption is *Causal Sufficiency*, which assumes that no other unobserved variable exists that influences any other pair of our set of variables, either directly or indirectly. We need this assumption because it is impossible to ensure that we have measured all possible variables (Pearl, 2009). The second assumption is the *Causal Markov Condition*. This condition dictates the relationship between process *X* and its associated graph *G*. It implies that once we know the value of a node's parent at time τ, all other variables in the past become irrelevant for predicting the state of the current node (Spirtes et al., 2000). The third assumption is *Faithfullness*. *Faithfullness* guarantees that the graph entails all conditional independence relations that are implied by the Markov condition (Spirtes et al., 2000).

Subsequently for the Causal Markov condition to hold true, the assumption that there is no *instantaneous (contemporaneous) causal effects* is needed. It may seem counterintuitive to consider the instantaneous effect between dynamical systems because the physical speed of information transfer, i.e., speed of light, is finite. The problem arises when the time-series cannot be sampled with sufficient resolution (Runge, 2018).

The causal discovery algorithm used in this framework is PCMCI. This approach was implemented in this framework to address some of the shortcomings of the PC (Peter and Clark) algorithm (Spirtes and Glymour, 2016). The PC algorithm was invented for random variables without assuming a time order (Lauritzen, 1996). Its process consists of several phases where first an undirected graphical model is estimated, then its links are adjusted using a set of logical rules (Spirtes and Glymour, 2016).

Tigramite defines the time-series graph of a stationary multivariate discrete-time stochastic process **X** of dimension *N* as graph structure G=(V×ℤ,E) of **X** where the set of nodes in the graph consists of the set of components *V* at each time *t* ∈ ℤ. The links in graph G are defined as a connection between variables $X_{t-\tau }^{i}$ and $X_{t}^{j}$ connected by a lag-specific directed link $"X_{t-\tau }^{i}\to X_{t}^{j}"\in G$ *for* τ > 0 *if and only if*

(1) Xt-τi ⫫ Xtj|Xt-\{Xt-τi},

where ${\text{X}}_{t}^{-}=\left({\text{X}}_{t-1},{\text{X}}_{t-2},\ldots \right)$. **X**, **X**_*t*_, and ${\text{X}}_{t}^{-}$ are considered as sets of random variables. The symbol \ denotes set difference (Runge, 2018).

The *stationarity* is assumed for process **X**. The process **X** is casually stationary over a time index set T *if and only if for all links* $X_{t-\tau }^{i}\to X_{t}^{j}$ *in graph* (Runge, 2018)

(2) Xt-τi ⫫ Xtj|Xt-\{Xt-τi} holds for all t∈T.

The framework constructs a time-series graph of a multivariate stochastic process **X**_*t*_ by evaluating the conditional mutual information (CMI) from subprocesses *X*_*t*−τ_ to *Y*_*t*_ for τ > 0

(3) $$\begin{array}{l} I\left(X_{t-\tau }^{i};Y_{t}|{\text{X}}_{t}^{-}\backslash \left\{X_{t-\tau }\right\}\right), \end{array}$$

with infinite past ${\text{X}}_{t}^{-}=\left({\text{X}}_{t-1},{\text{X}}_{t-2},\ldots \right)$. If *Y* ≠ *X*, the link *X*_*t*−τ_ → *Y*_*t*_ is considered as a *coupling or cross-link at lag* τ. If *Y* = *X*, then the link is considered an *autodependency or autolink at lag* τ (Runge, 2015).

The CMI for multivariate random variables *X*, *Y*, *Z* is defined as

(4) $$\begin{array}{l} I_{X;Y|Z}=\int \int \int dxdydz\;p(x,y,z)log\frac{p(x,y|z)}{p(x|z)\cdot p(y|z)},\\ \;=H_{X|Z}+H_{Y|Z}-H_{Z}-H_{X,Y|Z}, \end{array}$$

where *H* denotes the Shannon entropy and densities *p*(·) are assumed to exist (Runge, 2017). The framework tests the conditional independence hypothesis

(5) $$\begin{array}{l} H_{0}:X⫫Y|Z, \end{array}$$

against the general alternative. *I*_*X*; *Y*|*Z*_ = 0 if, and only if, *X* ⫫ *Y*|*Z*, provided that densities are well-defined (Runge, 2017). Tigramite utilizes a permutation-based generation of the distribution under *H*_0_ for hypotheses testing in graph structure construction. The conditional independence testing used in this framework is CMI, as defined in Equation (4) and is a model-free method, therefore, in principle, it can handle non-linear dependencies (Runge, 2018).

The framework then measures information transfer from the past of a process *X* at times *t*′ < *t* to the target variable *Y* at time *t* and excludes common information in history shared by *X* and *Y*. TE is defined as (Runge, 2015)

(6) $$\begin{array}{l} I\left(X_{t}^{-};Y_{t}|{\text{X}}_{t}^{-}\backslash X_{t}^{-}\right), \end{array}$$

(7) $$\begin{array}{l} I\left(X_{t}^{-};Y_{t}|{\text{X}}_{t}^{-}\backslash X_{t}^{-}\right)=\overset{\infty }{\underset{\tau =1}{\sum }}I\left(X_{t-\tau };Y_{t}|{\text{X}}_{t}^{-}\backslash X_{t}^{-},X_{t-\tau }^{-}\right), \end{array}$$

To overcome the *curse of dimensionality* of the condition in each term, TE is estimated using decomposed transfer entropy (DTE) (Runge et al., 2012), utilizing the theory of graphical models (Lauritzen, 1996; Eichler, 2012) which implies that

(8) $$\begin{array}{l} I\left(X_{t-\tau };Y_{t}|{\text{X}}_{t}^{-}\backslash X_{t}^{-},X_{t-\tau }^{-}\right)=I\left(X_{t-\tau };Y_{t}|S_{Y_{t},X_{t-tau}}\right), \end{array}$$

for a certain finite subset $S_{Y_{t},X_{t-tau}}\subset {\text{X}}_{t}^{-}\backslash X_{t}^{-}\cup X_{t-\tau }^{-}$ of the conditions. The suitable set $S_{Y_{t},X_{t-tau}}$ can be determined from the constructed time-series graph. The DTE is calculated by

(9) $$\begin{array}{l} I_{X\to Y}^{TE}\approx I_{X\to Y}^{DTE}=\overset{\tau^{*}}{\underset{\tau =1}{\sum }}I\left(X_{t-\tau };Y_{t}|S_{Y_{t},X_{t-tau}}\right), \end{array}$$

where τ^*^ is the smallest chosen τ (Runge et al., 2012).

The conditional independence test needed to compute CMI and TE in Tigramite is CMIknn, based on conditional mutual information estimated with the k-nearest neighbor entropy estimator developed by (Kraskov et al., 2004)

(10) $$\begin{array}{l} {\hat{I}}_{XY|Z}=\text{Ψ}\left(k\right)+\frac{1}{n}\overset{n}{\underset{i=1}{\sum }}\left[\text{Ψ}\left(k_{i}^{z}\right)-\text{Ψ}\left(k_{i}^{xz}\right)-\text{Ψ}\left(k_{i}^{yz}\right)\right] \end{array}$$

with the logarithmic derivative of the Gamma function $\text{Ψ}\left(x\right)=\frac{d}{dx}ln\Gamma \left(x\right)$. Free parameter *k* is the number of nearest neighbors in the joint space of X ⊗ Y ⊗ Z around each sample point *i* at maximum norm distance ϵ_*i*_. The $k_{i}^{xz}$, $k_{i}^{yz}$, and $k_{i}^{z}$ are computed by the number of points with a distance smaller than ϵ_*i*_ in the subspace X ⊗ Z, Y ⊗ Y, and Z to get $k_{i}^{xz}$, $k_{i}^{yz}$, $k_{i}^{z}$, respectively (Runge et al., 2017).

The appropriate maximum time delay τ_*max*_ usually depends on the nature of the signal being investigated. We can estimate the τ_*max*_ by observing the lagged unconditional dependencies decay. In this study, we observed that the dependencies decay beyond a lag of 15. For the significance level α, in the context of this framework it takes the role of a regularization parameter for model-selection, since precise assessment of uncertainty is not possible in iterative hypothesis testing. In our motor task-fMRI application, the algorithm parameters we used are as follow: maximum time lag τ_*max*_ = 15 time point, significance level α = 0.01 (Student's *t*-test).

To quantify causal interaction between the subprocess, this framework proposed a measure *I* to quantify linear causal effect (CE) of perturbation (Runge, 2015)

(11) $$\begin{array}{l} I_{i\to j}^{CE}\left(\tau \right)={\text{Ψ}}_{ji}\left(\tau \right) \end{array}$$

where Ψ(τ) is iteratively computed matrix products of estimated coefficient matrices Φ(τ) by (Runge et al., 2015)

(12) $$\begin{array}{l} \text{Ψ}\left(\tau \right)=\overset{\tau }{\underset{s=1}{\sum }}\text{Ψ}\left(s\right)\Phi \left(\tau -s\right). \end{array}$$

The mediated causal effect (MCE) through a component *k* is the sum over the products of path coefficients only along causal paths through *k*.

(13) $$\begin{array}{l} I_{i\to j}^{MCE}\left(\tau \right)={\text{Ψ}}_{ji}\left(\tau \right)-{\text{Ψ}}_{ji}^{\left(k\right)}\left(\tau \right) \end{array}$$

where Ψ^(*k*)^(*t*) is a computed from Equation (12) with modified path coefficient matrices Φ^(*k*)^(*t*) where all links toward component *k* are set to zero

(14) $$\begin{array}{l} {\text{Ψ}}_{ki}^{\left(k\right)}\left(\tau \right)=\{\begin{array}{l} 0,\;for\;all\;links\;X_{t-\tau }^{i}\to X_{t}^{k}\\ \Phi_{ki}\left(\tau \right),otherwise \end{array} \end{array}$$

which blocks all paths through component *k* at any lag (Runge et al., 2015).

Aggregated causal effect (ACE) and aggregated causal susceptibility (ACS) measures on the lag with maximum effect (Runge et al., 2015):

(15) $$\begin{array}{l} I_{i\to j}^{CE,\max}=\underset{0<\tau \leq \tau_{max}}{\max}|I_{i\to j}^{CE}\left(\tau \right)| \end{array}$$

(16) $$\begin{array}{l} I_{i\to j}^{ACE}=\frac{1}{N-1}\underset{j\neq i}{\sum }I_{i\to j}^{CE,\max} \end{array}$$

(17) $$\begin{array}{l} I_{i\to j}^{ACS}=\frac{1}{N-1}\underset{i\neq j}{\sum }I_{i\to j}^{CE,\max}. \end{array}$$

The average mediated causal effect (AMCE) is calculated based on causal paths through a given node

(18) $$\begin{array}{l} I_{k}^{AMCE}=\frac{1}{\left|C_{k}\right|}\underset{\left(i,j\right)\in C_{k}}{\sum }\underset{0<\tau \leq \tau_{max}}{\max}|I_{i\to j|k}^{MCE}\left(\tau \right)| \end{array}$$

where $C_{k}$ is the set of interactions between all non-identical pairs *i*, *j* ≠ *k* at all lags 0 < τ ≤ τ_*max*_ where *k* is an intermediate component (at any lag) and $\left|C_{k}\right|$ denotes its cardinality (Runge et al., 2015).

## 3. Results

### 3.1. Brain Activity Localization

While the localization of the motor-related brain region has been well studied, we performed univariate group subtraction analysis on this data to verify that this dataset is consistent with previous findings.

**Figure 2:**
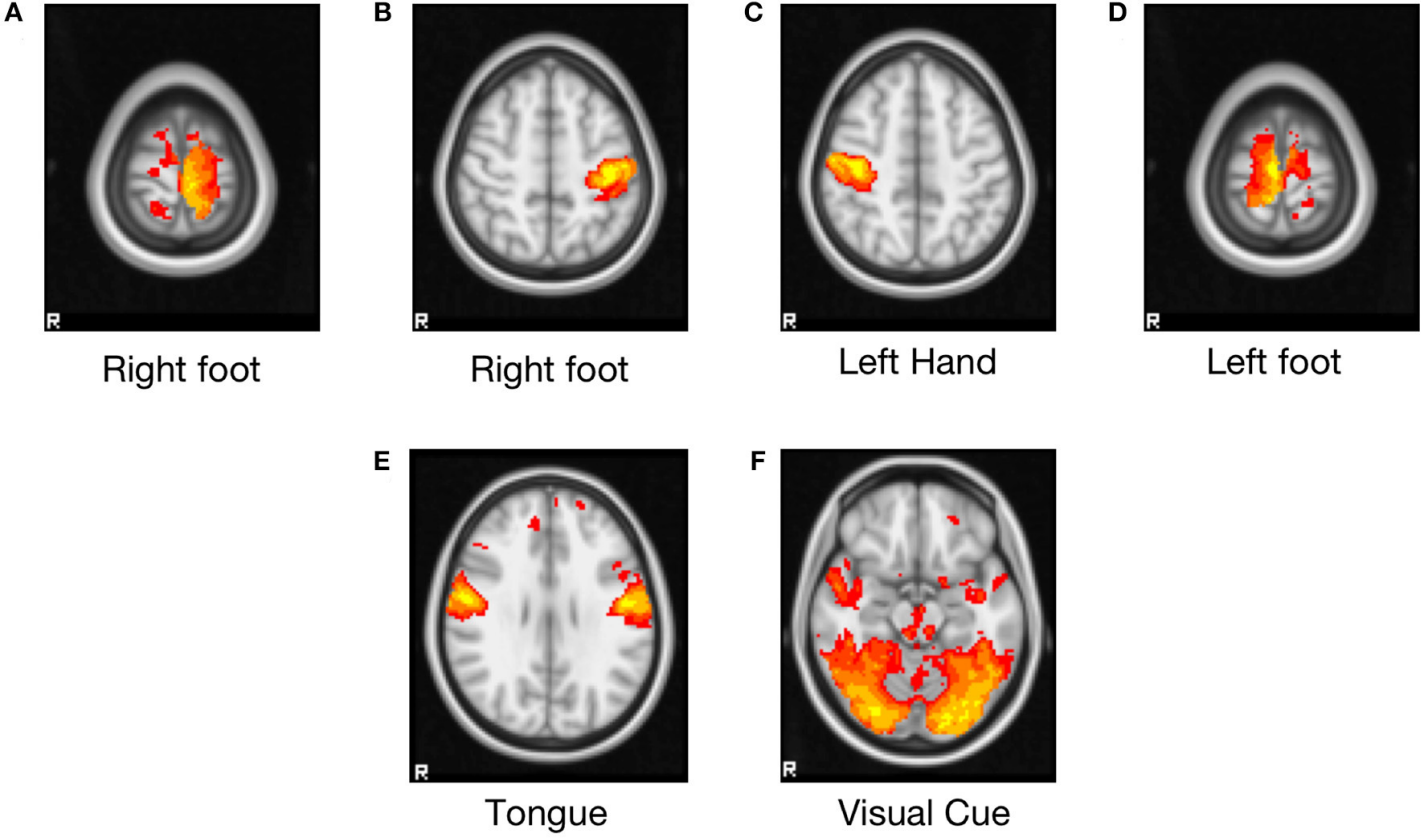
Group-level clusters of each task block from 50 randomly selected subjects of the HCP motor task-fMRI dataset. Z statistic images were thresholded using clusters determined by Z > 3.1 at a cluster significance threshold of *P* = 0.05 (corrected). FEAT (FMRI Expert Analysis Tool, v6.00) was used for the analysis. The General Linear Model was used to model six blocks (five task blocks, one cue block). Task blocks consisted of tapping left or right fingers, squeezing left or right toes, or moving the tongue, preceded by a visual cue block. The brain areas covered by these clusters are listed in Table 2. **(A)** Right foot, **(B)** right hand, **(C)** left hand, **(D)** left foot, **(E)** tongue, **(F)** visual cue.

The resulting main cluster of all the task blocks is shown in Figure 2, and the areas covering the activated brain region are listed in Table 2. The left and right foot tasks show clusters at the right and left premotor cortex (Brodmann area 6), respectively. The left- and right-hand tasks show clusters at the right and left primary motor cortex (Brodmann area 4). The tongue block shows clusters in both the left and right primary motor cortex. The visual cue block shows large cluster covers both in the left and right visual cortex V1 (Brodmann area 17) and V2 (Brodmann area 18).

**Table 2:** Area identified by univariate group subtraction as defined by Juelich histological atlas.

**Area name**	**Abbreviation used in this paper**
Left anterior primary motor cortex Brodmann area 4	PmcBA4aL
Right anterior primary motor cortex Brodmann area 4	PmcBA4aR
Left posterior primary motor cortex Brodmann area 4	PmcBA4pL
Right posterior primary motor cortex Brodmann area 4	PmcBA4pR
Left premotor cortex Brodmann area 6	PcBA6L
Right premotor cortex Brodmann area 6	PcBA6R
Left visual cortex V1 Brodmann area 17	V1BA17L
Right visual cortex V1 Brodmann area 17	V1BA17R
Left visual cortex V2 Brodmann area 18	V1BA18L
Right visual cortex V2 Brodmann area 18	V1BA18R

### 3.2. Causal Effect

**Figure 3:**
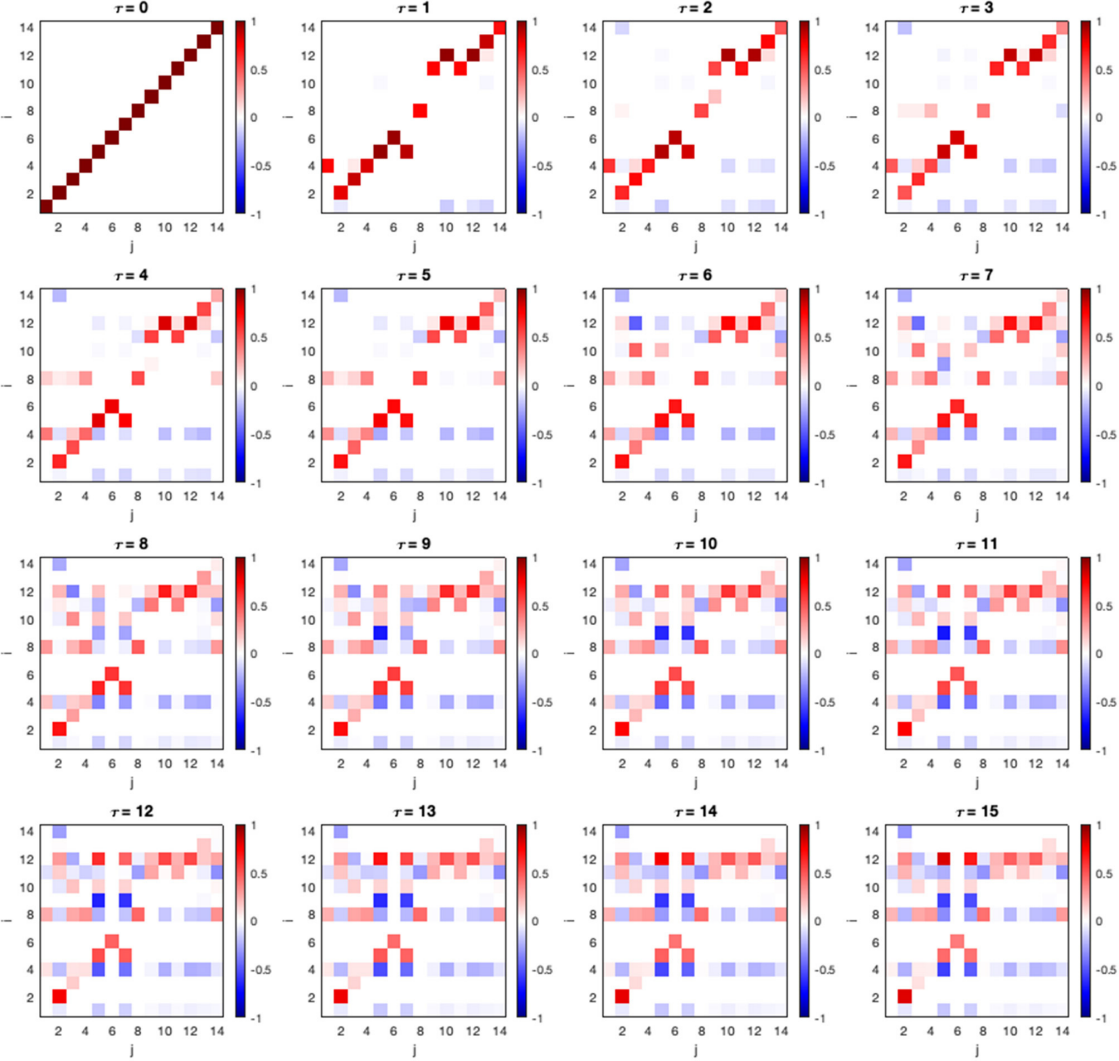
Causal effect of all ROI pairs and lags. An entry in the matrix shows causal effect $I_{i\to j}^{CE}\left(\tau \right)$ calculated using Equation (12) where *i* and *j* correspond to the ROI listed in Table 1. The strength declines in the longer lags.

In the following section, we show a constructed connectivity model and its associated parameter of a random HCP subject. In this framework, in addition to studying causal effects between adjacent nodes in the connectivity model, we can also study total causal effect (CE) along indirect causal paths. The matrices of CEs between all ROIs are shown in Figure 3. The CE between two components *i* and *j* at lag τ can be denoted as $I_{i\to j}^{CE}\left(\tau \right)$.

**Figure 4:**
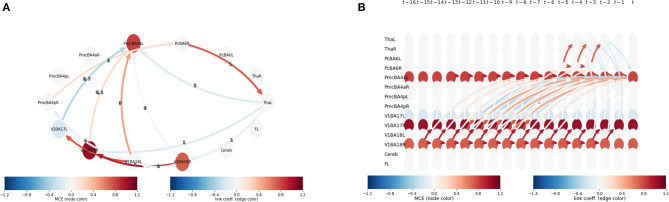
**(A)** An aggregated graphical model from V1BA18R to PmcBA4aL at lag 15 $I_{12\to 5}^{CE}\left(15\right)$. It is a summary graph representing the time-series graph in **(B)**. The edge color shows link coefficient and node color shows the MCE. **(B)** A time-series-graph of V1BA18R to PmcBA4aL pair at lag 15 $I_{12\to 5}^{CE}\left(15\right)$ depicts links in relevant causal paths between V1BA18R and PmcBA4aL at lag 15. The edge color shows link coefficient and node color shows the MCE.

We can observe that CE becomes stronger as the lag (τ) is increased at node 12, 8, and 4 or area V1BA18R, PmcBApR, and PcBA6R (Table 1), respectively. From this observation, we can further investigate the interaction between these areas by plotting their mediation graph. The Figure 4 is a mediation graph between V1BA18R and PmcBA4aL. We choose to investigate this pair because it has the highest CE, $I_{12\to 5}^{CE}\left(15\right)=0.82$. The time-series graph in Figure 4B shows the effects propagated from V1BA18R to other visual areas in the early process, then the strongest CE propagates to PmcBA4aL in the left hemisphere, and weaker CE propagates to PmcBA4aR in the right hemisphere. After that, the CE propagates from PmcBA4aR to PcBA6R. The left Thalamus only receives CE from PcBA6R and shows negative CE to several visual areas and PmcBA4aL. The negative CE means that it counteracts the effect of other areas.

### 3.3. Causal Gateway and Mediators

By calculating average causal effect (ACE), a column-mean of the CE matrices, and average causal susceptibility (ACS), a row-mean of the CE matrices, we can observe how much effect a specific ROI has on the rest of the brain and how sensitive a specific ROI is to perturbations from other parts of the brain. An average mediated causal effect (AMCE) measures how strong a subprocess mediates CEs propagating throughout the system (Runge et al., 2015).

**Figure 5:**
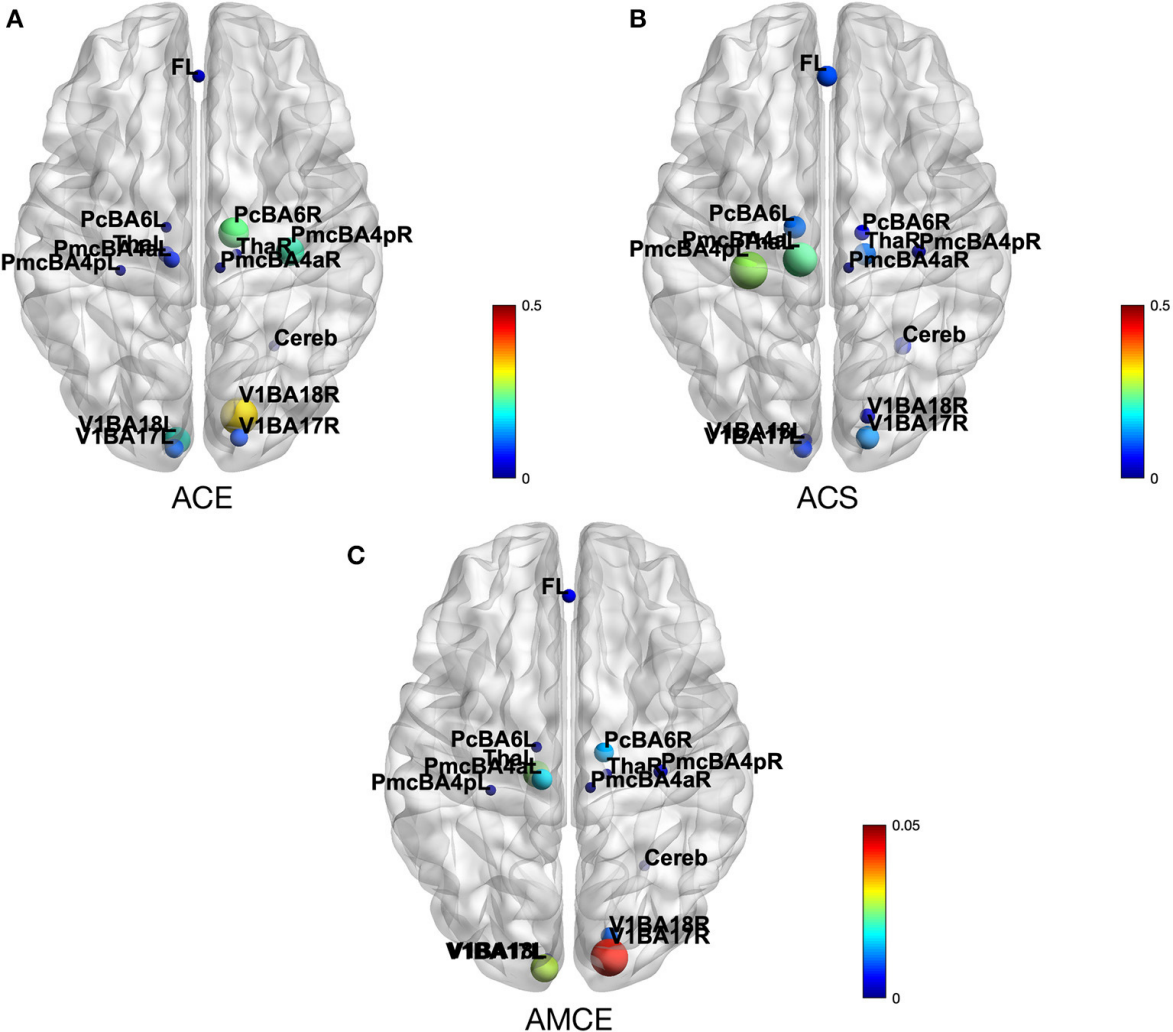
**(A)** Depicts the average causal effect (ACE) of each brain region (nodes) in HCP motor task-fMRI connectivity model. The values (size of the nodes) reflect how much a particular region effects the rest of the brain. **(B)** Average causal susceptibility (ACS) shows how sensitive the region is to the change from the rest of the system. **(C)** Average mediated causal effect (AMCE) shows how strong the region mediates the effect propagation.

In Figure 5, we show ACE, ACS, and AMCE for this HCP subject. The Figure 5A shows that this particular subject's right visual cortex (Brodmann area 18) has a strong effect on the rest of the brain. This is reasonable since in the HCP motor task-fMRI protocol, the subject's actions were initiated by a visual cue. In Figure 5B, we can see that the left primary motor cortex (Brodmann area 4) has high susceptibility with the change from other areas. We can infer the reason behind this observation to be due to the fact that this task focuses on motor function, thus most changes in the system affect motor-related areas the most. In Figure 5C, the right visual cortex (Brodmann area 17) is shown to be the strongest mediator of the CE spreading. This area acts as the main pathway to this system, corresponding to the experiment paradigm where their visual cue initiates the action.

## 4. Discussion

### 4.1. Connectivity Model Inference and Interpretation

Correctly interpreting brain mechanisms is difficult, especially in higher cognitive functions such as memory or self-awareness, because there is no ground truth, and some cognitive functions cannot be physically observed. In this study, we show that in addition to conventional connectivity model construction approaches such as Granger causality, Tigramite is also a viable approach with its own benefits. We chose to show its application with a motor task-fMRI dataset because mechanisms of brain motor function are well studied. The available knowledge can be used to compare and verify the validity of the resulting connectivity model.

The brain area involved in controlling the body's voluntary movement is the motor cortex. This area can be further divided into the primary motor cortex (Brodmann area 4), the premotor cortex (Brodmann area 6), and a supplementary motor area (Meier et al., 2008). The primary region of the motor system is the primary motor cortex. It works in association with the rest of the motor area to control muscle movement. Visual area 1 (V1), or Brodmann area 17 (BA17) in the visual cortex, functions primarily in pattern recognition in the visual field (Goetz, 2007). It processes visual information in association with other regions inside the visual cortex, such as Broadmann area 18 (BA18). The frontal cortex in the frontal lobe has been found to play roles in mediating movement-related brain signals (McFarland and Haber, 2002) through the thalamus and cerebellum (Bosch-Bouju et al., 2013). According to this knowledge, we used Tigramite to construct a connectivity model involving these regions.

The Tigramite framework shows not only the statistical dependency between brain regions, but also the causal effects among the regions. In addition to the topological information of the resulting graphical model, ACE, ACS, and AMCE provide information about the overall characteristic of the interaction inside the model.

ACE shows how much of an effect a ROI has on the rest of the brain. The ACE of the random subject depicted in the results section shows that the major driver of the system is the right visual area (BA17), followed by the right premotor cortex (BA6), and the right primary motor cortex (BA4), respectively (Figure 5A). It is reasonable to expect the visual cortex to be a major driver to the system, due to the fact that the motor task performed by the subject was initiated by a visual cue. Inside the motor cortex, ACE shows that the premotor cortex is the major driver to the system. It reflects the fact, found by studying brain activity of monkeys, that the premotor cortex is involved in planning and preparing for movement, after which the movement is then executed by the primary motor cortex (Weinrich and Wise, 1982).

The level of ACS shows how susceptible a ROI is to the perturbations from other parts of the brain. In our sample case, the brain areas with high ACS are the left primary motor cortex (BA4) and the left premotor cortex (BA6) (Figure 5B). Considering that the right motor cortex is shown to be a major driver, the high susceptibility of the left motor cortex may be evidence of inter-hemispheric coupling in brain activity, observable even during a uni-lateral motor task (Darvas and Hebb, 2014).

AMCE measures the subprocess that mediates the propagation of CEs throughout the system. Figure 5C demonstrates that the most dominant causal mediators is the right visual area (BA17), which means that this region is a major causal pathway of this system. The presence of dominant mediation and a driver in the right hemisphere may be related to the handedness of the subject. Unfortunately, the HCP dataset does not include this information in subject's profile, so we cannot confirm this conjecture.

In Figure 4, we show a connectivity model of the pathway between the right visual area (BA18) and the left primary motor cortex (BA4) at lag 15. While we expected a mediation pathway in the frontal lobe, caused by motor movement planning activity (Andersen and Cui, 2009), we could not detect it. The absence of this connection might be due to the fact that if the CEs are faster than the lag resolution, the repetition time (TR) in the case of fMRI—which is 2.8 s for this dataset—will appear in the analysis as contemporaneous links, which are not regarded as causal links (Runge et al., 2015).

### 4.2. Computational Cost and Consistency

The computational complexity of the PCMCI used in Tigramite depends on the complexity of the condition selection step and the momentary condition independence (MCI) test step. The complexity of the conditional selection step depends on the network structure where, the worst case being complete graph, the number of conditional independence tests for *N* variables are

(19) $$\begin{array}{l} N\overset{N\tau_{max}-1}{\underset{p=0}{\sum }}N\tau_{max}=N^{3}\tau_{max}^{2} \end{array}$$

tests with iteratively increasing cardinality (Runge et al., 2017). The number of tests in the MCI step are $N^{2}\tau_{max}$ tests for τ > 0 of a maximal dimensionality of $2+|\hat{P}\left(X_{t}^{j}\right)|+|\hat{P}\left(X_{t-\tau }^{i}\right)|$ where P denotes the causal parents (Runge et al., 2017). Thus, the overall complexity of the PCMCI is $N^{3}\tau_{max}^{2}+N^{2}\tau_{max}$ which is polynomial. The current implementation of the PCMCI does not yet support parallelization, however, it is a planned feature. There are several processes that have potential for parallelization, so the framework's performance may improve in the future.

Consistency is a property of the causal discovery method, indicating whether the method is able to converge to the true causal graph in the limit of the infinite sample size (Runge, 2018). Consistency of PCMCI is proven by Runge et al. (2017).

### 4.3. Advantages and Disadvantages of Tigramite

Graph theory is a classical tool used for brain function modeling, usually based on pairwise association measures among nodes in the graph (Bullmore and Sporns, 2009). In contrast, Tigramite uses CE which is a dynamical and causal alternative to classical measures, and which has been found to have a higher predictive power (Runge et al., 2015). The problem such as common driver, where *X* and *Y* are driven by a common *Z* process with a difference in time lag, reduces the validity and interpretability of the connectivity model in the framework where only a correlation between two variables is considered at a time, such as Granger causality (Mannino and Bressler, 2015). The implementation of Tigramite allows for a more detailed pathway analysis by investigating the CE of each node along lagged time. It mitigates the common driver problem by discovering hidden pathways and drivers.

However, limitations of this approach have to be considered when interpreting the model. This method is a data-driven approach; thus, it needs to rely on several assumptions. For example, causal sufficiency assumes that all variables are available and taken into account (Spirtes et al., 2000). When interpreting resulting CEs, it is important to consider it relative only to variables that were taken into account (Runge et al., 2015).

While we have shown that this approach is applicable on an individual level, to interpret brain mechanisms for the general population, a method to construct a group connectivity is needed because modeling connectivity from data across individuals is controversial (Duggento et al., 2018). Causal analysis is sensitive to temporal noise and variance. The variation in time-order of neuronal activity across individuals due to brain plasticity may cause misinterpretation of the causal effect. The fact that the BOLD signal is an indirect measurement of the actual neuronal activity further confounds the connectivity inference.

## 5. Conclusion

In this paper, we have shown the application of a novel time-lagged causal discovery, Tigramite (Runge, 2018), to a fMRI BOLD time-series to construct a brain connectivity model. We demonstrated the result using a motor task-fMRI dataset provided by the Human Connectome Project (Van Essen et al., 2012). We chose to use motor task-fMRI because the brain mechanism related to motor function has been well studied, so we could utilize this prior knowledge to compare it with the model constructed using this new approach. We have also shown the advantages of using this new approach. For example, this approach considers additional measurements (ACE, ACS, and AMCE) that are useful in understanding the dynamics of the model in addition to the topographical aspects of the connectivity. Moreover, the incorporation of time-lag into the analysis allows the discovery of both direct and indirect pathways, which the classical approaches may fail to identify. In the future, we hope that the benefits this framework provides may improve interpretability of more complex brain functions, such as, memory or consciousness, where the mechanism is difficult to observe, or in resting-state fMRI where there is no concrete driver to the system (Biswal, 2012). In such situations, understanding the characteristic of the model could improve the interpretation of the brain function of interest.

## Data Availability Statement

Publicly available datasets were analyzed in this study. This data can be found at: Human Connectome Project (https://www.humanconnectome.org).

## Ethics Statement

Ethical review and approval was not required for the study on human participants in accordance with the local legislation and institutional requirements. Written informed consent for participation was not required for this study in accordance with the national legislation and the institutional requirements.

## Author Contributions

SS carried out data processing and analysis. SS wrote the manuscript with support from YK. NY and YK helped supervise the project. SS, NY, and YK conceived the presented idea. All authors contributed to the article and approved the submitted version.

## Conflict of Interest

The authors declare that the research was conducted in the absence of any commercial or financial relationships that could be construed as a potential conflict of interest.
